# Children's thoughts and feelings about their donor and security of attachment to their solo mothers in middle childhood

**DOI:** 10.1093/humrep/dex016

**Published:** 2017-02-10

**Authors:** S. Zadeh, C.M. Jones, T. Basi, S. Golombok

**Affiliations:** 1 Centre for Family Research, University of Cambridge, Free School Lane, CambridgeCB2 3RQ, UK

**Keywords:** donor conception, solo mother, children's perspectives, attachment, mother–child relationships

## Abstract

**STUDY QUESTION:**

What is the relationship between children's thoughts and feelings about their donor and their security of attachment to their solo mothers in middle childhood?

**SUMMARY ANSWER:**

Children with higher levels of secure–autonomous attachment to their mothers were more likely to have positive perceptions of the donor, and those with higher levels of insecure–disorganized attachment to their mothers were more likely to perceive him negatively.

**WHAT IS KNOWN ALREADY:**

There is limited understanding of the factors that contribute to children's thoughts and feelings about their donor in solo mother families. In adolescence, an association was found between adolescents’ curiosity about donor conception and their security of attachment to their mothers.

**STUDY DESIGN, SIZE, DURATION:**

19 children were administered the Friends and Family Interview and Donor Conception Interview between December 2015 and March 2016 as part of the second phase of a longitudinal, multi-method, multi-informant study of solo mother families.

**PARTICIPANTS/MATERIALS, SETTING, METHODS:**

All children were aged between 7 and 13 years and had been conceived by donor insemination to solo mothers. Interviews were conducted in participants’ homes. The Friends and Family Interview was rated according to a standardized coding scheme designed to measure security of attachment in terms of secure–autonomous, insecure–dismissing, insecure–preoccupied and insecure–disorganized attachment patterns. Quantitative analyses of the Donor Conception Interview yielded two factors: interest in the donor and perceptions of the donor. Qualitative analyses of the Donor Conception Interview were conducted using qualitative content analysis and thematic analysis.

**MAIN RESULTS AND THE ROLE OF CHANCE:**

Statistically significant associations were found between the perception of the donor scale and the secure–autonomous and insecure–disorganized attachment ratings. Children with higher levels of secure–autonomous attachment to their mothers were more likely to have positive perceptions of the donor (*r* = 0.549, *P* = 0.015), and those with higher levels of insecure–disorganized attachment to their mothers were more likely to perceive him negatively (*r* = −0.632, *P* = 0.004). Children's narratives about the donor depicted him as a stranger (*n* = 8), a biological father (*n* = 4), a social parent (*n* = 3), or in ambivalent terms (*n* = 4).

**LIMITATIONS, REASONS FOR CAUTION:**

Findings are limited by the wide age range of children within a small overall sample size. Participants were those willing and able to take part in research on donor conception families. The statistical significance of correlation coefficients was not corrected for multiple comparisons.

**WIDER IMPLICATIONS OF THE FINDINGS:**

Findings highlight the importance of situating children's ideas about the donor within family contexts. It is recommended that those working with donor conception families consider this when advising parents about whether, what and how to tell children about donor conception.

**STUDY FUNDING/COMPETING INTEREST(S):**

This study was funded by the Wellcome Trust [097857/Z/11/Z]. The authors have no conflicts of interest to declare.

## Introduction

There has been a worldwide increase in the number of single women accessing fertility treatment with donated gametes (De Wert *et al*., 2014). Most of the empirical research on this topic has focused on single women who used donor sperm in their path to parenthood. This growing literature has generally shown that solo mother families do not differ in terms of family functioning or child adjustment from those headed by opposite-sex or same-sex couples (Chan *et al*., 1998; Murray and Golombok, 2005a,b; Golombok *et al*., 2016).

Less is known about how parents and children in solo mother families think and feel about the sperm donors involved in family creation. Regarding mothers’ perspectives, there is inconsistent evidence about the relationship between the significance attributed to the donor and the information known about him. In the US, anonymous donors have been found to be a symbolic presence within families (Hertz, 2006), while in the UK, it seems that some mothers prefer not to think about the donor, irrespective of whether he is anonymous or identifiable (Zadeh *et al*., 2016a). In general, it appears that single mothers may be more likely than their partnered counterparts to have shared, or plan to share, information about donor conception with their child (Scheib *et al*., 2003; Freeman *et al*., 2016). Some studies have shown that single mothers incorporate the donor into their child's ‘birth narrative’ (Hertz, 2006), while others have highlighted a tendency amongst mothers to postpone sharing this information (Landau and Weissenberg, 2010), and some uncertainty about doing so at all (Freeman *et al*., 2016).

Most of what is known about children's perspectives in solo mother families has been derived from mothers’ reports. In general, it seems that in the preschool years, and as they start school, children may ask about the whereabouts of their father (Hertz, 2006; Landau and Weissenberg, 2010; Weissenberg and Landau, 2012; Zadeh *et al*., 2016b), but their thoughts and feelings about donor conception are less well understood. Research that has elicited the reports of donor-conceived children in other family types suggests that children may think about their origins in multiple ways. Regarding donor information, Vanfraussen *et al*. (2001) found that of 41 children aged 7–11 years old with same-sex parents, 54% did not want more information, 19% wanted non-identifying information, and 27% wanted identifying information about the donor. Children with opposite-sex parents have also been shown to vary in their feelings, with Blake *et al*. (2014) finding that at age 10, children may feel positive, neutral, mixed or negative about being donor-conceived.

In studies of adolescents, thoughts and feelings about the donor have been found to vary according to family type. Those in solo mother families appear less likely than their two-parent counterparts to report feeling confused when first told, and more likely to refer to the donor as their ‘father’ or ‘biological father’, to feel positively about their donor conception, and to be curious about the donor, specifically, his reasons for donation, and his likeness to them (Scheib *et al*., 2005; Jadva *et al*., 2009; Beeson *et al*., 2011; Nelson *et al*., 2013). Differences between adolescents’ thoughts and feelings about the donor in solo mother and opposite-sex two-parent families have primarily been explained in terms of either father absence (Beeson *et al*., 2011), or the age at which children are told about donor conception (Jadva *et al*., 2009). However, most studies of adolescents in solo mother families have relied upon questionnaire methods, and have either recruited participants via online forums for those interested in making connections with the donor or children conceived using the same donor (Jadva *et al*., 2009, 2010; Beeson *et al*., 2011; Persaud *et al*., 2016), or studied adolescents whose donors are identifiable (Scheib *et al*., 2005).

More recently, researchers have investigated the other family factors that may contribute to adolescents’ thoughts and feelings about the donor, focusing on variation within, rather than between, family types. Slutsky *et al*. (2016) investigated the relationship between adolescents’ security of attachment to their parent(s) and their perceptions of the donor. According to attachment theory, relationships with caregivers influence psychological, emotional and social adjustment throughout the life course (Bowlby, 1988). Secure attachment patterns are based on the perception of caregiver(s) as a ‘secure base’ from which to explore the world and a ‘safe haven’ in times of stress; that is, the perceived availability of caregiver(s) to provide both instrumental and emotional support. Such patterns have been shown to positively impact upon children's outcomes at different developmental stages (Belsky and Cassidy, 1994). Conversely, insecure attachment patterns are associated with negative outcomes over time (Hesse and Main, 2000). In Slutsky *et al*.’s (2016) study the Friends and Family Interview (FFI) (Steele and Steele, 2005), an interview technique designed to assess children's security of attachment to their parents in middle childhood and adolescence, was administered to 19 donor-conceived adolescents in solo mother and same-sex two-parent families. An association was found between attachment security and curiosity about donor conception, with adolescents with secure attachment patterns reporting greater acceptance of their origins.

By using both quantitative and qualitative methods, this study sought to investigate whether attachment security was associated with thoughts and feelings about donor conception among pre-adolescent children in solo mother families. It explored how children in solo mother families think and feel about their donor conception and the donor at an age at which they appear to understand their donor conception (Blake *et al*., 2014) and are able to offer comprehensive information about their relationships with their caregiver(s) (Kriss *et al*., 2012). It is also the first study to have investigated the narratives about the donor told by pre-adolescent children in solo mother families.

## Materials and Methods

### Participants

Participants were interviewed as part of a longitudinal study of single mother families created by donor insemination in the UK. All families were originally recruited through one of the UK's largest fertility clinics that has also provided the longest-standing programme for single women. A random sample of single mother families with a donor-conceived child aged between 4 and 9 years was selected by the clinic, and invited to take part in the study. A participation rate of 72% was obtained. Families were previously visited when children were on average 5.7 years of age (Golombok *et al*., 2016; Zadeh *et al*., 2016b). At the present phase of the study, which is ongoing, families were visited in child age order, with families with the oldest children being revisited first. A total of 24 families were contacted, and 23 agreed to take part, giving a participation rate of 96%.

Interviews were conducted with 19 children aged between 7 and 13 years (mean = 10.3, SD = 1.82). Four children were not interviewed because they had not yet been told about the donor's role in their conception (*n* = 3), or did not consent to participation (*n* = 1). All participants had been told about their donor conception, and the majority (*n* = 16, 85%) were told this information before the age of 3; the remainder were told at ages 4 (*n* = 1, 5%), 8 (*n* = 1, 5%) and 12 (*n* = 1, 5%). Most children (*n* = 14, 74%) were conceived using an anonymous donor; the remainder (*n* = 5, 26%) were conceived using an identifiable donor. Ten children (53%) were male and 9 (47%) were female. Twelve children (63%) were singletons, five (26%) had one sibling also conceived through donor insemination. Two children (11%) had two siblings, one conceived naturally and one conceived through donor insemination. Four mothers (21%) were now in relationships, three (16%) of which were cohabiting, although all stated that they remained single parents.

### Procedure

Approval for the study was obtained from the University of Cambridge Psychology Research Ethics Committee. Written, informed consent was obtained from all participants and their mothers. One of three trained researchers (S.Z., C.M.J. or T.B.) interviewed children on their own at home. Each interview lasted ~1 h in duration. Interviews were transcribed and anonymised, and imported into the qualitative software program Atlas.ti.

### Measures

#### Donor Conception Interview

Children were administered a modified version of a semi-structured interview (Blake *et al*., 2014) designed to assess children's thoughts and feelings about being donor-conceived. Children were asked about their understanding of, and initial feelings about, donor conception, to describe what they imagined their donor to be like, their thoughts and feelings about him, if they discussed him with other people, if they perceived him as a family member, and if they had any questions they would like to ask him. Interviews were analysed both quantitatively and qualitatively.

Four variables were created from the interview schedule: (i) ‘description of donor’, (ii) ‘feelings about donor’, (iii) ‘thoughts about donor’ and (iv) ‘questions for donor’, and rated according to a standardized coding scheme. Description of donor and feelings about donor were each rated on a three-point scale ranging from ‘1’ negative through ‘2’ neutral to ‘3’ positive. Thoughts about donor was rated on a three-point scale from ‘1’ no through ‘2’ sometimes to ‘3’ yes. Questions for donor was rated on a four-point scale ranging from ‘1’ no questions through ‘2’ questions about the conception process and ‘3’ questions about the donor to ‘4’ questions about a relationship with the donor. Rating reliability for the ‘description of the donor’ and ‘feelings about the donor’ scales was ensured by the qualitative content analyses described below.

##### Quantitative analyses

Pearson correlation coefficients were calculated to examine relationships between the four variables. A significant positive association was found between description of donor and feelings about donor (*r* = 0.72, *P* < 0.001) and between thoughts about donor and questions for donor (*r* = 0.53, *P* = 0.02). Based on these findings, two scales were created. The first, perception of the donor, comprised description of donor and feelings about donor. The second, interest in the donor, comprised thoughts about donor and questions for donor. Higher scores on these scales indicated more positive perceptions of, and greater interest in, the donor, respectively.

##### Qualitative analyses

Qualitative content analyses (Mayring, 2000) conducted independently by two raters (C.M.J. and S.Z.) ascertained children's level of understanding about their donor conception and their feelings when first told. To provide greater insight into children's narratives about the donor, each transcript was analysed using thematic analysis (Braun and Clarke, 2006). A total of 18 codes across transcripts were generated by the first rater (C.M.J.). All codes and corresponding text segments were abstracted from the full text and re-read, and, where appropriate, codes were collapsed. Two main themes and four subthemes relating to children's perceptions of the donor were identified and cross-checked, first against the coded abstracts, and second, against the entire dataset. Following a second reading of all transcripts (S.Z.) and a systematic data audit (Flick, 2014), themes were refined, and a thematic map produced. Raters independently reviewed each transcript against the thematic map, identifying each child's donor narrative as corresponding to one of four subthemes. Transcripts that were not uniformly categorized (*n* = 5) were discussed until total agreement was reached.

#### Friends and Family Interview

Children were administered a modified version of the FFI (Steele and Steele, 2005), a semi-structured interview that asks about family members, friends, teachers and school experiences, and is designed to assess security of attachment in middle childhood and adolescence. The FFI has been shown to have good interrater reliability and construct validity (Kriss *et al*., 2012) and has been used successfully with both adopted and donor-conceived samples (Stievenart *et al*., 2012; Slutsky *et al*., 2016).

Interviews were coded using the FFI Rating and Classification System (Kriss *et al*., 2012) on several dimensions including truth, reflective functioning skills, and perception of mother as available to provide both practical and emotional support. Each of these constructs was rated on a four-point scale ranging from 1 (no evidence) to 4 (significant evidence). Individual scales were used to produce ratings on a four-point scale for each of the four global attachment classifications: (1) secure-autonomous, (2) insecure–dismissing, (3) insecure–preoccupied and (4) insecure–disorganized. Responses were coded with an emphasis on how the mother–child relationship was discussed. Interview transcripts were coded twice, first by one of the researchers (C.M.J.) and second, by an independent rater unaware of children's responses to the Donor Conception Interview. Interrater reliability for each attachment classification was calculated using Pearson correlations as follows: secure-autonomous (0.78), insecure–dismissing (0.71), insecure–preoccupied (0.84), insecure–disorganized (0.82), reflecting high levels of interrater agreement for the four attachment classifications. The first coder's ratings were used in all analyses.

##### Quantitative analyses

Pearson correlations between children's attachment ratings as determined by the FFI and children's scores on the perception of the donor and interest in the donor scales were conducted to examine the relationship between attachment and children's perceptions of and interest in the donor.

## Results

### Children's understanding of donor conception

Overall, 14 children (74%) were able to explain at least some aspect of donor conception; the remainder (*n* = 5, 26%) were unable or unwilling to do so. Explanations ranged from describing the fertility clinic or hospital, to mentioning the donor, to details about the conception process:

There's a special part in the hospital where you can go if you don't have a boyfriend … so you can just go ask … if people volunteer to help make a baby. (11-year-old boy)

I was made by a donor, that's about all I know. (9-year-old girl)

To make a baby you need sperm from a man and an egg from a woman and some very kind men helped my Mum make me and they donated their sperms. (9-year-old girl)

### Children's memory of, and responses to, disclosure

Twelve children (63%) reported remembering being told about their donor conception, and 9 (48%) recalled initial responses to this information: feeling different (*n* = 1, 5%), confused (*n* = 3, 16%), surprised (*n* = 1, 5%), neutral (*n* = 2, 11%) or positive (*n* = 2, 11%):

I didn't really know what my Mum was talking about. I thought she was talking gibberish. (10-year-old boy)

I just remember accepting it and moving on. (12-year-old girl)

Of the seven children (37%) who could not remember being told, three (16%) reported that they had always known.

### Children's thoughts, feelings and descriptions of the donor

Approximately half of the children (*n* = 10, 52%) reported that they did not think about the donor. Six children (32%) said they thought about him only occasionally, and three (16%) reported that they did think about him:

Yeah, before I heard the description of him, I always imagined him to have black hair, a moustache, blue eyes, that's how I always imagined him. (8-year-old boy)

Children's descriptions and feelings about the donor varied (Table I).

**Table I:** Description of and feelings about the donor. Data are *n* (%).

	Negative	Neutral	Positive
Description of the donor	3 (16)	5 (26)	11 (58)
Feelings about the donor	2 (10)	11 (58)	6 (32)

Negative descriptions included:

He's just a weird man who helped to make babies, that's it. (8-year-old girl)

I don't really know [what sort of person he is]. I don't really care. Probably not a trustworthy one … because of his job. (12-year-old girl)

Negative feelings included:

I really don't think he'd be the best Dad because if he doesn't even want to see his own child then I don't think he'd be the best. (9-year-old girl)

I just feel like I want to be with him, want him to live with us. (8-year-old boy)

Positive descriptions included:

He's probably quite a kind person … the sort of person who wants to help people. (11-year-old girl)

I think he would be kind, he would be caring, he would be nice, maybe loving. (8-year-old boy)

Positive feelings included:

I kind of admire him I suppose. (11-year-old girl)

I feel that he helped me a lot to be alive. (9-year-old girl)

### Children's questions for the donor

Most children (*n* = 15, 79%) had at least one question for the donor (Table II).

**Table II:** Questions children would like to ask the donor.

Question topic	Number of children who would like to ask about this	Examples
No questions^a^	4	
Age	4	*‘I would ask when he was born, how old he is.’*
Reasons for donation	4	*‘Why did he feel like that was a good thing to do?’*
Donor–child relationship^b^	3	*‘Would you ever like to be my actual dad?’*
Occupation	3	*‘Just ask about his job and that's it.’*
Personality/interests^c^	3	*‘I'd like to know about his personality and stuff like that, what he likes to do.’*
Children conceived using same donor	2	*‘Where do my other brothers and sisters live?’*
Conception process^d^	2	*‘What type of medicine did you have to give mummy?’*
Donor's family	2	*‘Is he married to someone else and does he have children that he knows?’*
Appearance	1	*‘What does he look like?’*
Name	1	*‘What his name is.’*

^a^Example of ‘no questions’.

^b^Example of ‘questions about a relationship with the donor’.

^c^Example of ‘questions about the donor’.

^d^Example of ‘questions about the conception process’.

#### Children's narratives about the donor

More than half (*n* = 11, 58%) of children's donor narratives drew upon notions of biological and social parenthood. Some children (*n* = 4, 21%) distinguished between the donor's role as a biological contributor and the role of a social parent, while others appeared to either confuse (*n* = 4, 21%) or entirely conflate (*n* = 3, 16%) these roles. Conversely, several children (*n* = 8, 42%) described the donor in minimal terms, as neither a biological contributor nor social parent. Narratives thus corresponded to one of four subthemes on a continuum: (i) donor as stranger, (ii) donor as biological father, (iii) ambivalence towards donor and (iv) donor as social parent.

#### Donor as stranger

These narratives were generally brief. They did not include a description of the donor as either a biological or social parent, and did not identify him as family:

He's got no role in my life, so, sort of just, he's not part of my family or anything … He just feels like another person in the world. (11-year-old girl)

Some narratives were positive, describing the donor as ‘kind’ and ‘helpful,’ while others were either neutral or negative. For some children, answering questions about what the donor might be like seemed challenging:

I've no idea who they are … I actually wonder who he is. (13-year-old boy)

I don't know what he's like because I've never met him. (8-year-old girl)

Other children stated that they did not have a father:

It's just a guy's characteristics and parts apparently … Whenever [other people] ask I just say ‘don't have a Dad’. (12-year-old boy)

#### Donor as biological father

Narratives about the donor as a biological father often emphasized his physical characteristics:

He has dark features, dark eyebrows, eyes … Mum says you have his eyes or hair. And we wouldn't have these kind of features if our Dad was blonde. (12-year-old boy)

Apparently it was a man that was tall, that's probably why we're tall. (12-year-old girl)

One child also described the donor's personality on the basis of her own:

I think he's quite a lively and musical person who enjoys going outside and running around and being all crazy and whatever because that's a bit like me. (12-year-old girl)

These narratives generally described the donor as ‘technically my Dad in a way’, ‘sort of’ or ‘kind of like family’. One child mentioned using information provided by the fertility clinic when discussing the donor:

If people ask something about my Dad … They ask ‘Do you know him?’ and I probably say ‘no’ or something like, ‘but I have a sheet that says about him.’ (10-year-old boy)

#### Ambivalence towards donor

Ambivalent narratives often included multiple interpretations of the donor, and mixed feelings about him and his status as a father:

I felt fine [when first told] because I was used to not having a Dad and I didn't really care … I wish I could just meet him … because he's my Dad after all. (13-year-old girl)

It's normal for me because I'm just used to not having a Dad … I don't really want to meet him because if I do I'll probably just miss him, because I guess he is kind of my Dad. (11-year-old boy)

Children were also ambivalent as to whether or not the donor was family:

I'd describe him as a half Dad like he's half not my Dad but he half is my Dad. (11-year-old boy)

My Dad … He's not really family, maybe genetically or biologically whatever you say, not really my family. (10-year-old boy)

In one narrative, mixed feelings about the donor were discussed in relation to making contact with him in the future:

Mum said that one day when I'm older I could find my biological Dad … I feel quite happy because I kind of like not having a Dad. (10-year-old girl)

In another, ambivalence was highlighted in relation to donor siblings:

I think there are eight others so far that are the same as me, same Dad … He was quite kind because … he wanted to make life even though he didn't want to see us for some reason. I can't think of him really … I don't know if I would like him or hate him if I did see him … he would have to be shared out between about 13 other kids, so. (10-year-old boy)

#### Donor as social parent

Narratives in this category were likely to mention the possibility of a social relationship with the donor, or the current lack thereof, with multiple feelings expressed:

I don't really think he'd be the best Dad because he doesn't want to be … I'd still call him Dad. (9-year-old girl)

I know what I feel like but I also don't know because I really want to meet him someday but I also don't … because I'm not sure what he'll be like and yeah, I'm not sure if he will want to see me. (9-year-old girl)

These narratives were also likely to describe the donor as family. Two referred to donor siblings:

Now you have to be 18 to see him, but when I'm 18 I want to see him and I want to meet some of my half-brothers and sisters. (9-year-old girl)

[I'd ask him] ‘Where do my other brothers and sisters live? And are you going to live here?’ (8-year-old boy)

### Relationship between attachment and children's thoughts and feelings about the donor

Pearson correlation coefficients were calculated between children's attachment ratings on each of the four dimensions of the FFI coding scheme and children's scores on the perception of the donor and interest in the donor scales to examine the relationship between attachment security and children's perceptions of, and interest in, the donor (Table III). A significant positive association was found between the secure-autonomous scale of the FFI and the perception of the donor scale (*r* = 0.549, *P* = 0.015), showing that children with higher levels of secure-autonomous attachment had more positive perceptions of the donor. In addition, a significant negative association was found between the insecure–disorganized scale of the FFI and the perception of the donor scale (*r* = −0.632, *P* = 0.004), reflecting more negative perceptions among children with higher levels of insecure–disorganized attachment.

**Table III:** Pearson correlations between the Friends and Family Interview attachment scales and the ‘Interest in the donor’ and ‘Perception of the donor’ scales from the Donor Conception Interview.

	Secure-autonomous	Insecure-dismissing	Insecure-preoccupied	Insecure-disorganized
Interest in the donor	0.097	−0.374	0.369	0.073
Perception of the donor	0.549^*^	−0.085	−0.110	−0.632^**^

* Correlation is significant at the *P* < 0.05 level (2-tailed).^**^Correlation is significant at the *P* < 0.01 level (2-tailed).

## Discussion

Findings suggest that in middle childhood, children in solo mother families are diverse in their thoughts, feelings and narratives about the donor; while some children feel positively, others are neutral or negative in their evaluations. Additionally, although some children do not appear to have thoughts about or questions for the donor, others seem curious about him. Children's questions for the donor ranged from those focussed on the possibility of a relationship, to wanting identifying, or non-identifying, information. These findings—both about the diversity of children's perceptions of the donor, and their differing levels of curiosity about him—are in keeping with what is known about the perspectives of donor-conceived children at a similar developmental stage in both same-sex and opposite-sex two-parent families (Vanfraussen *et al*., 2001, 2003; Blake *et al*., 2014).

This study also investigated the relationship between children's ideas about the donor and their patterns of attachment to their mothers. In middle childhood, children with secure-autonomous attachments continue to perceive their caregivers as both reliable and responsive to their needs, while those with insecure–disorganized attachments exhibit behaviours that indicate that a consistent strategy towards their caregivers is lacking (Bosmans and Kerns, 2015). Children with higher levels of secure-autonomous attachment were found to be more likely to have positive perceptions of the donor, and those with higher levels of insecure–disorganized attachment were more likely to perceive him negatively. It should be noted, however, that no correction for multiple comparisons was carried out. Like Slutsky *et al*.’s (2016) study, the present investigation suggests that attachment patterns may be of predictive value in understanding how young donor-conceived people will think and feel about the donor. That these findings are shared across two separate studies of two separate samples, one in the USA and the other in the UK, and one of adolescents and the other of younger children, incites further, in-depth investigation of larger samples to increase understanding of the association between the nature of mother–child relationships and thoughts and feelings about the donor over time.

Children's interviews were also analysed qualitatively, illustrating that for some children, the donor remains a stranger about whom little is known or thought, while for others, he is a biological relative, who may be responsible for some of their traits. Some children seem to find positioning the donor difficult, and thus describe him in ambivalent terms, as at once a biological and social parent. For a minority, the donor is primarily understood in a social role, and these children are likely to report wanting to meet him, and/or other children conceived using his gametes. Notions of biological and social parenthood are also found in the donor narratives of children in same-sex two-parent families (Van Parys *et al*., 2015). Although distinguishing between these two concepts seems to be more challenging for some children in solo mother families than it is for others, it is perhaps unsurprising that at the age at which children become embedded in peer networks, they draw upon dominant narratives of family life in order to make sense of their conception.

Understanding the factors that contribute to particular narratives about the donor among the present sample is challenging. While previous research has established that age at disclosure is important for perceptions of the donor and donor conception (Jadva *et al*., 2009, 2010; Hertz *et al*., 2013), the majority of children interviewed were told about their donor conception at an early age. However, it is noteworthy that more of the younger children referred to the donor as a social parent than did those who were older, perhaps because younger children may be more likely to understand family relationships on the basis of frequency of contact (Perlesz *et al*., 2006) than are older children. Those who are younger are also be more likely to have been conceived with identifiable donors, owing to the UK Human Fertilisation and Embryology Act (2008). Whether or not younger children are more likely to conceptualize the donor as a social parent, and if and how this relates to donor identifiability is not yet known. This question should be prioritized as more of the children in the study reach the required age for follow-up (≥7 years).

It seems clear that for some children, ideas about the donor are shaped by information shared by their mothers. Findings may thus be said to reflect the power asymmetry of parent–child relationships at this developmental stage (Van Parys *et al*., 2015), as children are not yet of an age at which they can independently attempt to access information about the donor or other children conceived using his gametes. Other research would seem to suggest that curiosity about these connections among those in solo mother families may continue into adolescence (Scheib *et al*., 2005; Jadva *et al*., 2009; Beeson *et al*., 2011), and it is worth noting that some of the children who believed they could identify the donor at age 18 were conceived using anonymous donors. Although very few children expressed a desire to meet the donor, findings thus highlight a need for both mothers and children to receive accurate information regarding the possibility of future contact (Zadeh, 2016). Further research would benefit from studying children's and mothers’ narratives simultaneously.

Although middle childhood remains an understudied developmental stage in research on attachment (Bosmans and Kerns, 2015), findings suggest that donor-conceived children's thoughts and feelings about the donor may be best investigated through a family systems approach (Kerr and Bowen, 1988) that accounts for the role of relationships and processes within families. Given the small sample size, and the relatively wide age range within it, conclusions must be drawn cautiously, yet findings are enhanced by the quantitative and qualitative analyses conducted. The study design made it possible both to gain in-depth information from children about their thoughts and feelings about the donor, and to assess their attachment relationships to their mothers. In general, the study highlights the importance of situating children's ideas about the donor within the family context. Future research with larger samples should examine this context in greater depth by investigating the role that siblings, other family members and significant others may play in children's donor, and wider family, narratives. It is recommended that those working with donor conception families consider advice relating to conversations about the donor within the context of existing parent–child relationships and family life more generally.
